# Eight-year study of the Staphylococcus epidermidis resistance profile against glycopeptides, oxazolidinones and glycylcyclines in an ICU of a Greek tertiary hospital

**DOI:** 10.1186/cc13545

**Published:** 2014-03-17

**Authors:** A Stylianakis, V Kaldis, D Argyris, D Chatzilia, V Kasouris, F Kalogeropoylos, S Kamariotis, K Daskalakis, S Stratoyli, I Alamanos

**Affiliations:** 1 General Hospital Kat-Eka, Kifisia Athens, Greece

## Introduction

*Staphylococcus epidermidis *(SE) is the most often isolated species of coagulase-negative staphylococci, which are recognized as one of the main causes of ICU infections [1]. In this study we aimed to study the resistance profile of SE clinical isolates against last-line antibiotics (vancomycin (VA), teicoplanin (TEC), linezolid (LZ) and daptomycin (DA)) for treating CNS infections, during an 8-year period.

## Methods

From January 2005 until December 2012 we examined 518 nonduplicated SE isolates recovered from blood cultures of 421 patients hospitalized in a surgical ICU of our hospital. Species identification and susceptibility testing were performed using the automated VITEK II system (Biomerieux). Additionally we used the E-test method (Biomerieux, ABI-Biodisk) in order to confirm some isolation resistances against TEC and LZ found by the VITEK II system and to estimate the MIC levels of DA and VA. Mueller-Hinton agar adjusted to contain physiologic levels of free calcium ions (50 μg/ml) was used when testing DA susceptibility. Isolates with MIC >4 mg/l were considered resistant to TEC and LZ and those with MIC <1 mg/l and MIC <4 mg/l susceptible to DA and VA, respectively.

## Results

The percentage resistance rate of the examined SE isolates is shown in Table 1. Methicillin resistance was observed with an overall prevalence of approximately 84.6%. All of the resistant isolates to TEC and LZ were also resistant to methicillin. The MIC values of VA were lower than 2 mg/l (Table 1).

**Table 1:** 

	2005 (*n *= 77)	2006 (*n *= 76)	2007 (*n *= 79)	2008 (*n *= 89)	2009 (*n *= 92)	2010 (*n *= 93)	2011 (*n *= 46)	2012 (*n *= 59)
VA	0	0	0	0	0	0	0	0
TEC	1.3	2.6	0	0	0	1.1	0	0
LZ	0	0	1.4	1.2	6.8	12.6	23.1	27.1
DA	Not tested	Not tested	Not tested	0	0	0	0	0

## Conclusion

The examined SE isolates present a scattered resistance to TEC and they show a remarkable continuing increase of resistance to LZ. These findings enforced the necessity to take the appropriate measures in the ICU environment and during the clinical practice to limit the dissemination and the amplification of these resistances. DA and VA possess an excellent *in vitro *activity against SE isolates and they could be very good alternative solutions for treating ICU infections caused by this species.
